# Effectiveness and Safety of Toripalimab Combination Therapies for Patients With Chemo-Resistant Choriocarcinoma

**DOI:** 10.3389/fonc.2022.815917

**Published:** 2022-04-14

**Authors:** Xiaomei Liu, Xiuqin Li, Hui Qu, Shiyue Zhang, Ruizhe Zhang, Zhenhua Du

**Affiliations:** ^1^ Department of Obstetrics and Gynecology, Shengjing Hospital of China Medical University, Shenyang, China; ^2^ Shanghai Junshi Biosciences Co, Ltd., Shanghai, China

**Keywords:** choriocarcinoma, chemo-resistant, PD-1 checkpoint inhibitors, toripalimab, effectiveness and safety

## Abstract

Toripalimab as a novel PD-1 inhibitor has presented its promising efficacy in patients who developed chemo-refractory carcinomas, whereas no study has ever investigated the effectiveness of toripalimab in chemo-resistant choriocarcinoma. Here we reported the effectiveness and safety data of 4 patients with chemo-resistant choriocarcinoma who underwent PD-1 antibody therapy by toripalimab and individualized chemotherapies. From January 2019 to August 2020, 4 patients with choriocarcinoma were admitted in Shengjing Hospital of China Medical University. The patients’ age ranged from 29 to 52 years with a median of 36 years. All the patients achieved CR after the combined therapy of toripalimab with individualized chemotherapies according to the decreased serum β-hcg level. Two of the four patients were observed with treatment-related adverse events (AEs), including one grade I skin rash and one grade I pruritus. Our cases showed that toripalimab combined with chemotherapy presented a tolerable safety profile and promising effectiveness in patients with chemo-resistant choriocarcinoma, indicating its potential as salvage therapy for this subset of patients.

## Introduction

Gestational trophoblastic neoplasia (GTN) represented a spectrum of rare tumors, including malignant invasive mole, choriocarcinoma, placental site trophoblastic tumor (PSTT), and epithelioid trophoblastic tumors (ETT), accounting for <1% gynecologic cancers (1). Among all types of GTN, invasive moles and choriocarcinoma have the highest global incidence, and choriocarcinoma is the most common type in Southeast Asia (2, 3). Currently, standard treatment with single-agent or poly-chemotherapy regimens can be effective in over 90% of choriocarcinoma (1). However, high toxicity in multidrug regimen, developed resistance to chemotherapy, frequent recurrency, and poor prognosis remained to be significant problems in some patients (4, 5). Therefore, novel therapeutic approaches are still warranted.

Programmed cell death receptor 1 (PD-1) and its ligands, programmed cell death ligand-1 (PD-L1), have been the most studied immune checkpoint proteins in these years. Recent studies revealed a promising effect of the PD-1 inhibitor by pembrolizumab as a novel solution for management of the targeting patients by reporting a series of cases with chemotherapy-resistant GTN (4, 6–9). Therefore, targeting PD-1 inhibitory signaling might yield clinical benefit for patients with chemotherapy-resistant or refractory GTN. Toripalimab, a humanized IgG4 monoclonal antibody against PD-1, is one of the first PD-1 inhibitors that were approved by the China Food and Drug Administration (CFDA) into clinical trials, which has demonstrated its manageable safety profile in several cancer types, such as urologic cancer and gastric cancer (10–12). In addition, toripalimab also presented the preliminary clinical activity in patients with chemo-refractory melanoma and malignant solid tumors (13, 14).

With enlightenment from the previous studies, immune checkpoint inhibitors combined with chemotherapy have become the standard treatment in some types of cancer. Our premise is that toripalimab combined with the standard chemo-regimens might provide a novel treatment option in the management of patients with chemotherapy-resistant choriocarcinoma. Yet, there were limited studies evaluating combination salvage therapies with the PD-1 inhibitor in chemotherapy-resistant patients with choriocarcinoma. Herein, we reported our findings from the treatment of a series of cases with choriocarcinoma which failed in previous standard chemotherapy and became chemotherapy-resistant.

## Case Presentation

### Ethics Approval and Consent to Participate

This study was approved by the ethics committee of Shengjing Hospital of China Medical University (2021PS552K). Written consent was obtained from participants.

### Case 1

**Figure 1:**
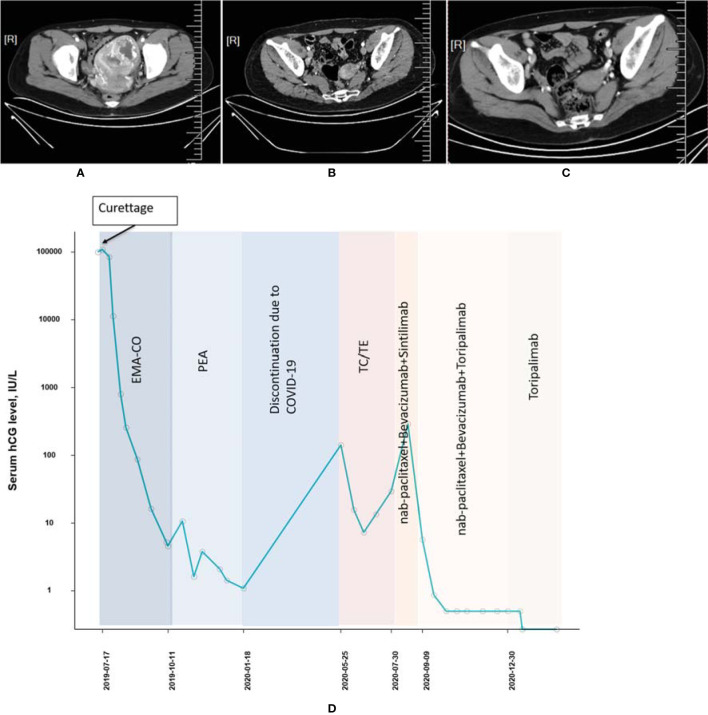
Computed tomography imaging **(A)** before chemotherapy, **(B)** before initiation of PD-1 inhibitor, and **(C)** after combined treatment of PD-1 and chemotherapies (patient 1). **(D)** The concentration of the β-hcg level since the diagnosis of choriocarcinoma (patient 1).

A 29-year-old woman (patient 1, obstetric status G2P0) with a history of vagina hemorrhage who underwent curettages twice was admitted to our hospital in July 2019. The initial serum β-hcg of the patient was 108,197 IU/l according to outpatient records, and thoracic CT imaging suggested a high possibility of pulmonary metastasis. The tumor was of stage III with a WHO prognostic score of 11 points. After a series of multiagent chemotherapies (EMA-CO × 6, PEA × 4, TC/TE [paclitaxel + carboplatin alternating with paclitaxel + etoposide] × 2) from July 2019 to July 2020, the tumor was eventually considered resistant to chemotherapy due to the elevated β-hcg level. Treatment with the PD-1 inhibitor was initiated, and CT imaging was conducted throughout the treatment period (**Figures 1A–C**). From July 2020 to September 2020, a regimen of nab-paclitaxel (200 mg, d1) combined with bevacizumab (300 mg, d1) and sintilimab (200mg) was applied to the patient twice. However, the β-hcg concentration did not fall to the normal range thereafter. Since September 2020, as sintilimab in the previous regimen was replaced by toripalimab (240 mg), β-hcg has decreased dramatically and become normalized, and complete response (CR) was finally achieved. From October 2020 to December 2020, the patient tolerated the treatment well after 7 cycles of consolidation regimens ([nab-paclitaxel (200 mg, d1) + bevacizumab (300 mg, d1) + toripalimab (240 mg)] × 4, [nab-paclitaxel (400 mg, d1) + bevacizumab (500 mg, d1) + toripalimab (240 mg)] × 2, toripalimab (240 mg)] × 1) with no AE observed (**Figure 1D**).

### Case 2

**Figure 2:**
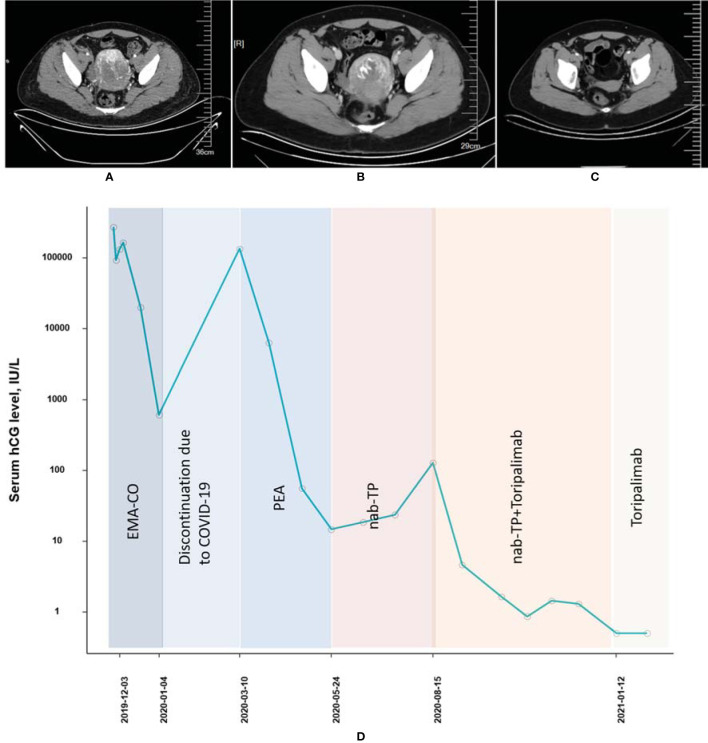
Computed tomography imaging **(A)** before EMA-CO chemotherapy, **(B)** after EMA-CO chemotherapy, and **(C)** after combined treatment of PD-1 and chemotherapies (patient 2). **(D)** The concentration of the β-hcg level since the diagnosis of choriocarcinoma (patient 2).

A 32-year-old woman (patient 2, obstetric status G2P0) who was diagnosed with choriocarcinoma and underwent 5 cycles of 5-fluorouracil + actinomycin-D before being admitted to our hospital in December 2019 was of stage III with a WHO prognostic score of 14 points. EMA-CO (3 cycles), PEA (4 cycles), and TP (paclitaxel + cisplatin, 2 cycle) were subsequently administrated from December 2019 to July 2020. During the period of chemotherapy, the patient had to suspend the treatment due to the COVID-19 epidemic in China from January 2020 to March 2020. The β-hcg concentration of this patient at admission was 271,746 IU/l and never fell to the normal range while receiving many lines of cytotoxic chemotherapies. In August and September 2020, the combined therapy of TP (paclitaxel 400 mg, d1, cisplatin 40 mg, d1–3) and toripalimab (240 mg) was initiated and applied twice, incorporated with 6 cycles of consolidation treatments ([TP + toripalimab] × 4, toripalimab × 2); the β-hcg level decreased to 1.64 IU/l in October 2020 and remained stable ever since (**Figures 2A–D**). As of yet, the patient did not complain pf any AE other than a grade I skin rash.

### Case 3

**Figure 3:**
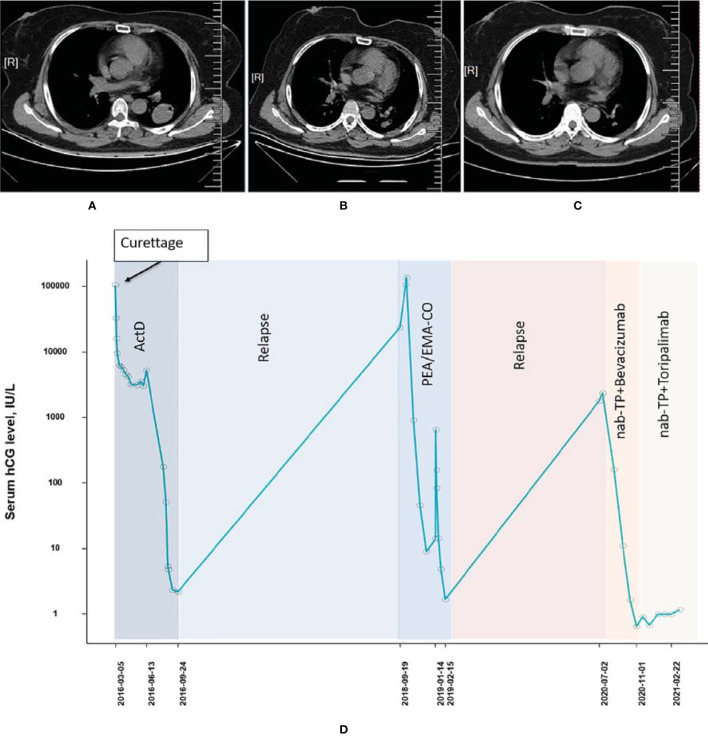
Computed tomography imaging **(A)** before pulmonary metastasis, **(B)** before combined treatment of PD-1 and chemotherapies, and **(C)** after combined treatment of PD-1 and chemotherapies (patient 3). **(D)** The concentration of the β-hcg level since the diagnosis of choriocarcinoma (patient 3).

The patient was a 52-year-old woman (patient 3, obstetric status G6P2) diagnosed with a hydatidiform mole in 2016. She had a history of 2 times of curettage and total abdominal hysterectomy while receiving a series of single-agent chemotherapies (actinomycin-D × 4) before the disease had progressed. The disease recurred and progressed to choriocarcinoma in September 2018 and presented with pulmonary metastasis during later examinations. In January 2019, the tumor was of stage III with a WHO prognostic score of 12 points. The patient underwent percutaneous radiofrequency ablation of lung metastases from choriocarcinoma and was subsequently treated with EMA-CO between January 2019 and March 2019. The tumor still relapsed in the pulmonary node even if the patient had undergone X-knife and radiotherapy treatment in May 2019. The combined chemotherapy of paclitaxel (400 mg, d1) and cisplatin (35 mg, d1–3) followed by a consolidation regimen of TP + bevacizumab (500 mg, d1) was administered to the patient from July 2020 to October 2020. The β-hcg level decreased steadily during the treatment and achieved biochemical CR, albeit radiological imaging showed an ongoing partial remission (**Figures 3A–D**). The disease status was stabilized by treating with 2 additional cycles of the TP regimen combined with toripalimab (240 mg). Currently, only a grade I pruritus was reported while receiving the PD-1 inhibitor treatment.

### Case 4

**Figure 4:**
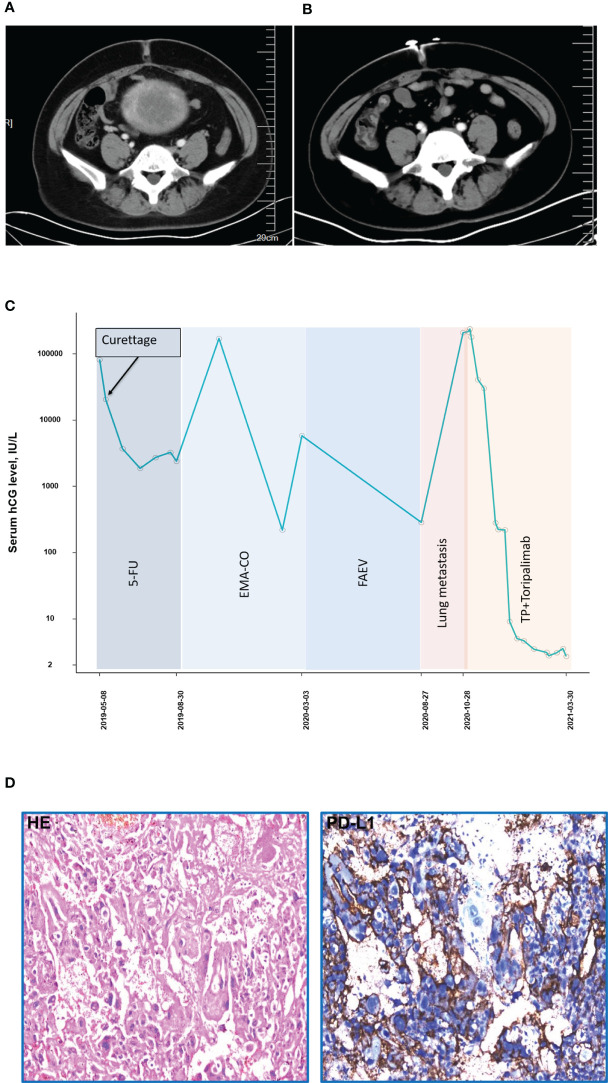
Computed tomography imaging **(A)** before combined treatment of PD-1 and chemotherapies **(B)** after combined treatment of PD-1 and chemotherapies (patient 4). **(C)** The concentration of the β-hcg level since the diagnosis of choriocarcinoma (patient 4). **(D)** Immunohistochemistry of PD-L1 expression (patient 4).

A 40-year-old patient (patient 4, obstetric status G8P2) complained of a previous diagnosis of stage III choriocarcinoma and a history of administration of numerous chemotherapy regimens (5-fluorouracil × 5, 5FU+ actinomycin-D + vincristine × 1, EMA-CO × 4, EMA-EP [etoposide + methotrexate + actinomycin-D alternating with etoposide + cisplatin] × 1, FAEV [fluorouracil + actinomycin-D + etoposide + vincristine] × 4) without showing any improvement. After being admitted to our institution in March 2020, the patient was diagnosed as stage III choriocarcinoma with a WHO prognostic score of 16 points, and the pulmonary metastasis was presented as well. The combined treatment was introduced in November 2020. The patient received 5 cycles of TP (paclitaxel 400 mg, d1, cisplatin 35 mg, d1–3) + toripalimab (240 mg) before the β-hcg level decreased to the normal range. CT imaging demonstrated an effective radiological response (**Figures 4A, B**). A CR was achieved, and 6 additional consolidation cycles ([TP + toripalimab] × 4, toripalimab × 2) were performed to maintain the disease status (**Figure 4C**). Immunohistochemistry of the tumor component for PD-L1 revealed positive PD-L1 expression (tumor proportion score = 30%, combined positive score = 31, **Figure 4D**) using the 22C3 antibody (Dako, Santa Clara, CA). A total of 35 gene mutations were detected by WES. The mutation genes mainly concentrated on the tumorigenic signaling pathways, ABC transporter signaling pathways, and extracellular matrix receptor interaction signaling pathways (**Figure S1**). The patient tolerated the treatment well and was followed until February 2021 with no adverse events observed.

## Discussion

While most of the patients with choriocarcinoma would achieve CR from single-agent chemotherapy, there were limited treatment strategies available for those who were resistant to chemotherapy. Given the chemo-sensitive nature of choriocarcinoma, an extremely limited number of patients might progress to chemo-resistant choriocarcinoma. Considering the rarity of the disease setting, we reported 4 patients with chemo-resistant choriocarcinoma in this article, and their treatment strategies before and after the disease progression (**Table 1**). Our study showed that toripalimab combination therapy might be beneficial for patients with chemo-resistant choriocarcinoma. The β-hcg of each patient remained negative during the subsequent treatments until the last follow-up date.

**Table 1:** Patient characteristics.

Patient no.	1	2	3	4
Age, year	29	32	52	40
Performance status	1	1	1	1
Obstetric status	G2P0	G2P0	G6P2	G8P2
Previous gestational status	Abortion	Abortion	Hydatidiform mole	Hydatidiform mole
Time from antecedent pregnancy, months	1	11	52	40
hCG at diagnosis, IU/L	108,197	271746	2367	209306
Site of disease	Pulmonary	Pulmonary	Pulmonary	Pulmonary
FIGO score at diagnose	(III:11)	(III:14)	(III:12)	(III:16)
History of surgery	Curettage	Curettage	Curettage, total abdominal hysterectomy	Curettage
Regiments of chemotherapy	EMACO *6 PEA *4 TC/TE *2 nab+Bev+PD1 *3	EMACO *3 PEA *4 TP *2 TP+PD1 *2	TP *1 TP+PD1 *1	TP+PD1 *5
Cycles before β-hcg became normalized	15	11	2	5
Consolidated treatment	nab+Bev+PD1 *4 PD1 *3	TP+PD1 *4 PD1 *2	TP+PD1 *4 PD1 *2	TP+PD1 *4 PD1 *2
Number of consolidated treatments	7	6	6	6
IT MTX	–	–	–	–
Radiological response	–	–	Ongoing PR	–
AE (grade)	–	Skin rash (1)	Pruritus (1)	–
Response	CR	CR	CR	CR
End of follow-up	2021/12/30	2021/12/30	2021/12/30	2021/12/30

PEA, cisplatin + etoposide + actinomycin-D; TP, paclitaxel–cisplatin; EMACO, etoposide + methotrexate + actinomycin-D + cyclophosphamide + vincristine G (gravida); Nab, albumin paclitaxel; EC, cyclophosphamide; PD-1, programmed cell death receptor 1; PEB, cisplatin + etoposide + bleomycin; TC/TE, paclitaxel + carboplatin alternating with paclitaxel + etoposide; Bev, bevacizumab; P, para; REECP, reactive cutaneous capillary endothelial proliferation; CR, complete response; PD, progressive disease; PR, partial response; IT: intrathecal.

Performance status was determined according to the Zubrod–ECOG–WHO score. Adverse events were graded according to the Common Terminology Criteria for Adverse Events (CTCAE) v4.03. Radiological responses were evaluated according to Response Evaluation Criteria in Solid Tumors (RECIST) v1.1. The asterisk represents the multiplication sign, and the combined number represents the number of courses.

Programmed cell death 1 (PD-1) is a transmembrane glycoprotein of the Ig superfamily that acts as a T-cell coinhibitory receptor (15). Blockade of the PD-1 pathway activity with antibodies against PD-1 has been investigated in choriocarcinoma with encouraging results (16, 17). Toripalimab as a novel PD-1 inhibitor has presented its promising efficacy in patients who developed chemo-refractory carcinomas (12, 18), whereas no study has ever investigated the effectiveness of toripalimab in chemo-resistant choriocarcinoma. To the best of our knowledge, we only found 4 studies which reported a total of 5 cumulative cases (**Table 2**) using pembrolizumab as the single PD-1 inhibitor in the treatment of chemo-resistant choriocarcinoma (4, 6, 19, 20). The results of these studies suggesting PD-1-directed therapies might provide a novel treatment option in this disease setting, which is congruent with the results of ours. However, as no standard treatment for chemo-resistant choriocarcinoma was recommended in current clinical practice, it should be noted that the patients in our institution were all treated with combined therapy of both individualized chemotherapeutic strategy and different PD-1 inhibitors (with or without sintilimab), which might not be conclusive in terms of the effectiveness of each treatment regimen. Nevertheless, the number of cycles from the initialization of PD-1 inhibitors to the first time of achieving normal β-hcg ranged from 1 to 10 cycles, which is almost consistent with the range of 1 to 11 cycles in previous studies. All suggested that PD-1-directed therapy by toripalimab might be a highly effective therapeutic option for those patients. Regarding the safety effect, only Choi et al. reported a grade II skin rash in one of their cases with chemo-resistance epithelioid trophoblastic tumor (ETT) (7). Despite that two of four cases in our study were observed with AEs during treatment, the severity and course of these events were manageable. The difference in the incidence and severity of AEs can possibly be explained by that we were using multidrug chemotherapies simultaneously with toripalimab, which might increase the burden for patients to tolerate the toxic effect from chemotherapy. In the analysis, we used toripalimab irrespectively combined with chemotherapy for our patients. Besides, despite that all patients recovered from chemo-resistant choriocarcinoma well and remained stable till the latest follow-up date, varieties of treatment-related AEs including skin rash and pruritus were observed. Although these AEs were manageable, the different types of treatment-related AEs implied the variety and complicated impact of each inhibitor on safety profile, which should be further investigated.

**Table 2:** Treatment response to PD-1 inhibitor in chemo-resistant choriocarcinoma by literature.

Age	WHO score	Previous cycles of CTx	PD-L1 expression (%)	No. of PD-1 treatment		Response	F/U after treatment (months)	Adverse events (grade)	Reference
				Cycles to serologic remission	Consolidation cycles				
26	18	#8	Strong	#2	#4	CR	\	Hepatotoxicity	(6)
42	17	#25	100	#4	#5	CR	>24	Arthralgia	(4)
37	6	#18	100	#2	#5	CR	>5	Neutropenia, synovitis	(4)
30	NA	#30	Strong	#10	N/A	CR	>31	NA	(19)
50	NA	#40	100	#3	#3	CR	>22	Neuropathy (3)	(20)

CTx, chemotherapy; CR, complete response; PD-L1, programmed cell death ligand 1; F/U, follow-up; NA, not applicable.

There are limitations in our analysis. Firstly, regardless of the rarity of chemo-refractory choriocarcinoma, this retrospective case series study reported the results from only 4 patients, and some patients discontinued their treatment owing to the COVID-19 pandemic. Another aspect is that the treatment regimen for patients with chemo-resistant choriocarcinoma has not been unified in our institution, leading to a complicated prognosis effect during the follow-up. In our defense, an explanation for this situation is the complexity of the prior disease courses the patients received before the administration of toripalimab; in addition, there is no standard protocol for the treatment in this disease setting. Furthermore, our institution has been trying to optimize the treatment effect by utilizing a combined therapy rather than the single-agent immune checkpoint inhibitor that has been investigated before. Based on the results from this analysis, a direct comparison between combined therapy and monotherapy of toripalimab with sufficient sample size is warranted in further investigation.

Combined therapy of toripalimab and chemotherapy might be a novel salvage therapy for patients with choriocarcinoma who were resistant to prior chemotherapies. Long-term follow-up and sustaining surveillance are required in future study to verify the prognostic effect.

## Data Availability Statement

The raw data supporting the conclusions of this article will be made available by the authors, without undue reservation.

## Ethics Statement

The studies involving human participants were reviewed and approved by ethic committee of Shengjing Hospital of China Medical University. The patients/participants provided their written informed consent to participate in this study.

## Author Contributions

XmL designed the study. XmL, XqL, HQ, and RZ analyzed and interpreted the patient data. SZ and RZ collected the data. XmL and ZD prepared the manuscript. ZD revised the manuscript. All authors contributed to the article and approved the submitted version.

## Conflict of Interest

Author SZ and RZ were employed by Shanghai Junshi Biosciences Co., Ltd.

The remaining authors declare that the research was conducted in the absence of any commercial or financial relationships that could be construed as a potential conflict of interest.

## Publisher’s Note

All claims expressed in this article are solely those of the authors and do not necessarily represent those of their affiliated organizations, or those of the publisher, the editors and the reviewers. Any product that may be evaluated in this article, or claim that may be made by its manufacturer, is not guaranteed or endorsed by the publisher.

## Supplementary Material

Supplementary Figure 1 Genetic landscape of whole-exome sequencing in Patient 4.
