# Supplementary figures and images for: Effectiveness and Safety of Toripalimab Combination Therapies for Patients With Chemo-Resistant Choriocarcinoma
Source: Front Oncol. 2022 Apr 14;12:815917. doi: 10.3389/fonc.2022.815917 (PMC9047865; doi:10.3389/fonc.2022.815917)

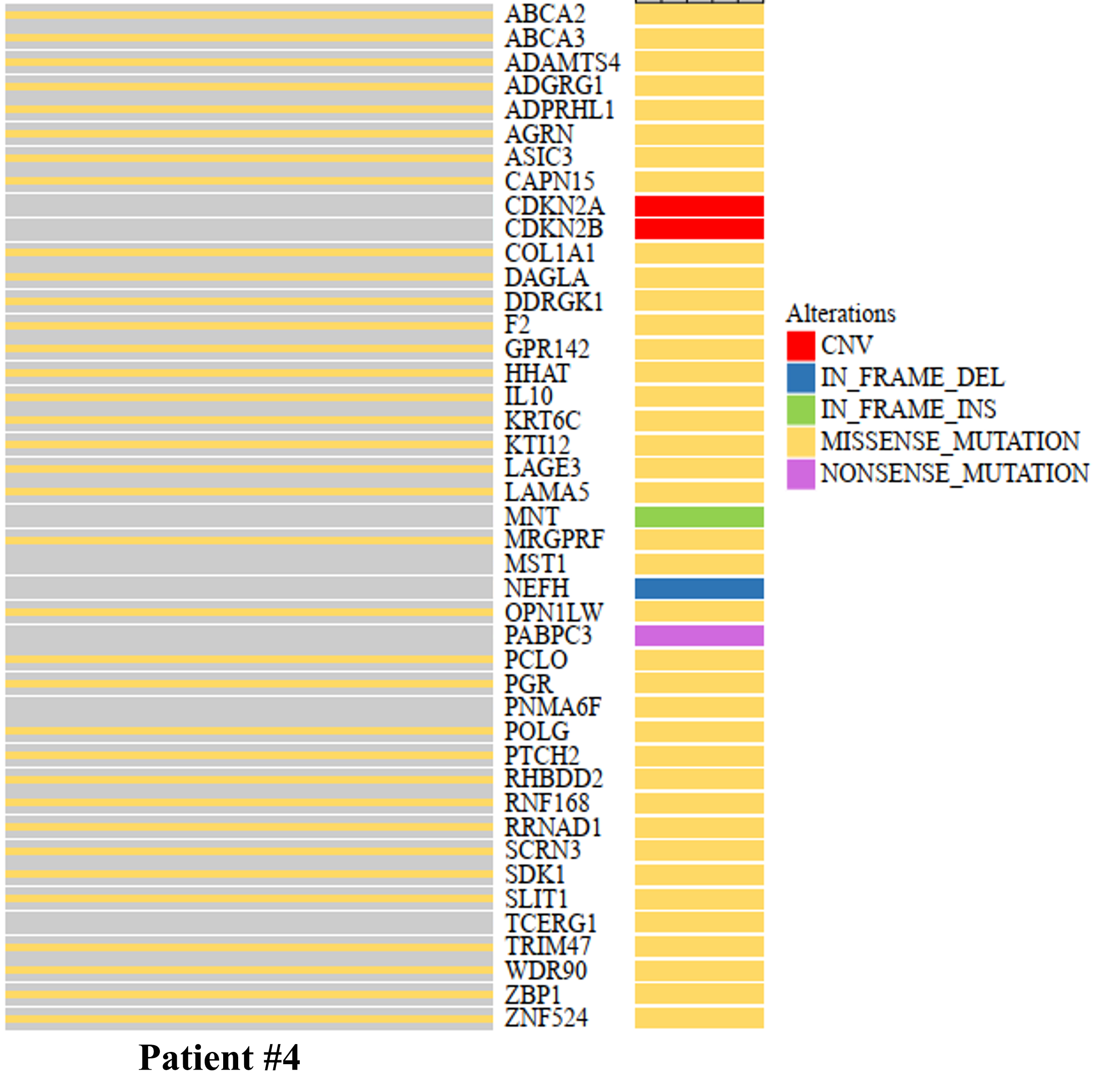

Supplement: Supplementary Figure 1 — Genetic landscape of whole-exome sequencing in Patient 4. [file Image_1.tif]
